# Breastfeeding trends, influences, and perceptions among Italian women: a qualitative study

**DOI:** 10.1080/17482631.2020.1734275

**Published:** 2020-02-27

**Authors:** Andrea L. DeMaria, Jaziel Ramos-Ortiz, Kelsie Basile

**Affiliations:** a Department of Public Health, Purdue University, West Lafayette, IN, USA; b Division of Consumer Science, Purdue University, West Lafayette, IN, USA; c School of Nursing, Purdue University, West Lafayette, IN, USA

**Keywords:** International health, Women’s Health, breastfeeding, social marketing, motherhood, social norms

## Abstract

**Purpose**: Breastfeeding behaviours are routinely assessed in worldwide capacities, and the World Health Organization (WHO) European Region has the lowest rates of exclusive breastfeeding. Rates in Italy are not well documented but suggest breastfeeding rates are rising since the early 2000s. Professional recommendations suggest exclusive breastfeeding should persist until the infant is at least six to twelve months of age. However, barriers to adhering to this recommendation exist, often resulting in a lack of initiation or premature cessation of breastfeeding behaviours. This study explored women’s perceptions, attitudes, and experiences with breastfeeding living in Florence, Italy.

**Methods**: Participants were 44 reproductive-aged (M = 31.7 ± 6.14; Range = 19 to 45 years) women currently utilizing the Italian healthcare system. All participants completed an in-depth, individual interview between June and August 2017 on topics related to reproductive health, including breastfeeding.

**Results**: Resulting themes relate to breastfeeding trends and influences, the role of identity and empowered choice, as well as perspectives on public breastfeeding.

**Conclusions**: Findings provide practical recommendations for future exploration and social marketing campaign application related to breastfeeding decision-making empowerment. Results can also be used for between-country comparison of breastfeeding behaviours and attitudes.

## Introduction

Breastfeeding behaviours are routinely assessed in worldwide capacities, and the World Health Organization (WHO) European Region has the lowest rates of exclusive breastfeeding, with less than 25% of infants exclusively breastfed within the first six months of life (World, Health Organization [WHO], 2015). Despite breastfeeding rates being reported among many European countries, breastfeeding information within Italy specifically is not well documented (Lauria, Spinelli, & Grandolfo, 2016). Available statistics report breastfeeding rates in Italy rising since the early 2000s, increasing from 81.1% in 2000 to 85.5% in 2013 (Italian National Institute of Statistics, 2013). Similarly, average breastfeeding duration rose from 6.2 months in 2000 to 8.3 months in 2013 (Italian National Institute of Statistics, 2013), aligning with professional recommendations (WHO, 2017).

In 1990, the Innocenti Declaration on the Protection, Promotion and Support of Breastfeeding, taking place in Florence, Italy, released an international call to action aiming to protect, promote, and support breastfeeding efforts (UNICEF, 1990). Produced by WHO, UNICEF, and other global government policymakers, the declaration encouraged and spread awareness of breastfeeding benefits for both mother and baby. In 2005, Florence, again, was the gathering place for the 15^th^ anniversary celebration of the Innocenti Declaration, where policymakers assessed breastfeeding support progress (UNICEF, 2005). Consequently, since the induction of the Innocenti Declaration, Italy has exhibited longstanding support for the promotion and encouragement of breastfeeding behaviours. Italy adheres to the WHO breastfeeding recommendations, promoting exclusive breastfeeding for approximately six months after an infant’s birth (WHO, 2017). The Italian Paediatric Societies issued their position statement in 2015 detailing various breastfeeding recommendations, including: consideration of breastfeeding as a nutritional norm, promotion and provision of education for mothers regarding the physiology of lactation, and the contraindication of breastfeeding only accepted for suitable medical reasons (Davanzo, Romagnoli, & Corsello, 2015). Following these recommendations ensures healthy physiological and psychosocial infant development, prevents infection, and reduces likelihood of childhood obesity, along with other mother and infant health benefits (Bagci Bosi, Eriksen, Sobko, Wijnhoven, & Breda, 2016). Policies and initiatives, such as the Baby-Friendly Hospital Initiative (BFHI), have also been enacted internationally to contribute to breastfeeding promotion and the health and wellness of mother and baby. BFHI, an evidence-based intervention meant to contribute to breastfeeding initiation, exclusivity, and duration, has been applied worldwide with the goal of helping nations achieve the breastfeeding gold standard (American Academy of Pediatrics, 2012; Cattaneo & Buzzetti, 2001; Kramer et al., 2001; WHO, UNICEF, 2003). However, a study assessing the effectiveness of BFHI in Italy, specifically, found that implementation of BFHI did not demonstrate a significant effect on the rates of breastfeeding, suggesting region-specific differences across breastfeeding interventions may require additional exploration (Cattaneo et al., 2016). Despite this, limited data does indicate Italy’s breastfeeding initiation and continuation rates remain high and continue to grow (Italian National Institute of Statistics, 2013; Lauria et al., 2016).

In general, breastfeeding is favourable among European women (Synnott et al., 2007). Italy-specific data are limited, warranting further exploration into this country’s cultural influences on the behaviour; however, studies conducted in the US and EU provide insight on breastfeeding motives. The aforementioned infant and mother health benefits are among the principal motivations for women’s breastfeeding behaviours as women often cite breastfeeding’s natural, healthy, and beneficial outcomes as correlates for initiation and sustainability (Kambale, 2011; Office of the Surgeon General, CDC, Office on Women’s Health, 2011; Sloan, Sneddon, Stewart, & Iwaniec, 2006). Breastfeeding promotion and sources of support also contribute to favourable attitudes towards breastfeeding. Clinical (i.e., doctor, other medical professional) recommendation has proved influential in women’s breastfeeding decision-making (Dieterich, Felice, O’Sullivan, & Rasmussen, 2013). When medical professional breastfeeding endorsement is coupled with breastfeeding support by family, friends, and partners, women are more likely to initiate and sustain breastfeeding behaviours (McFadden et al., 2017). Psychosocial factors impact breastfeeding choice (i.e., type of feeding women received from their mothers), along with the perception of breastfeeding as a social norm (Scott, Shaker, & Reid, 2004). The combination of a mother’s attitudes towards breastfeeding, social norms, and the opinions of trusted sources heavily dictate not only breastfeeding decision-making, but also actual breastfeeding behaviours (Scott et al., 2015). Similarly, mothers can be easily deterred from breastfeeding if they reside in certain areas where it is not accepted as the optimal method and other infant feeding approaches are the norm (Scott & Mostyn, 2003).

Despite the aforementioned, breastfeeding barriers do exist, restricting breastfeeding initiation or resulting in premature breastfeeding cessation. Employment issues are among the most significant barriers to breastfeeding in Italy (Kambale, 2011; Quintero Romero, Bernal, Barbiero, Passamonte, & Cattaneo, 2006). More specifically, research suggests intention to return to work, as well as full-time postpartum employment, result in lower breastfeeding initiation rates (Hawkins, Griffiths, Dezateux, & Law, 2007). Parental leave provisions are meant to improve mother and baby’s health and welfare. Italy’s parental leave policies vary for mothers and fathers: mothers are prescribed a mandated absence from work for a period of the pregnancy and post-natal recovery, while fathers are offered a five-day leave that can be redeemed at any point within five months of the child’s birth (Istituto Nazionale della Previdenza Sociale, 2019). While these policies can contribute to breastfeeding encouragement, mandatory maternity leave can also pose a threat to a mother’s participation in the labour market, creating a paradox between motherhood and career investment (Strang & Broeks, 2017). Media, social networks, and medical professionals’ strong push towards breastfeeding as the only or best option may also be problematic, resulting in feelings of guilt or failure if mothers decide against breastfeeding or experience breastfeeding difficulties (Hunt & Thomson, 2017; Taylor & Wallace, 2012; Thomson, Ebisch-Burton, & Flacking, 2015). This perception of breastfeeding as an “all or nothing” approach disregards the complex reality of infant feeding decision-making, limiting mothers and their autonomy (Brown, 2016). In many instances, these feelings of guilt or inadequacy may result in mothers engaging in early breastfeeding cessation or neglecting to initiate breastfeeding at all (Hunt & Thomson, 2017).

An additional breastfeeding barrier affecting women internationally (Amir, 2014; Office of the Surgeon General, CDC, Office on Women’s Health, 2011), including women living in Italy, is the perception of public breastfeeding behaviours. In Italy, there is no law against public breastfeeding; however, perceptions of public breastfeeding among Italian communities vary, with some perceiving it as embarrassing or taboo (Scott et al., 2015). The perceived disapproval yields feelings of embarrassment among mothers resulting in numerous avoidant strategies, such as extracting milk at home for on-the-go use, replacing breast milk with infant formula, or restricting and reducing time away from home (Scott et al., 2015). Although all viable methods, persistent engagement in the aforementioned avoidant strategies is not conducive to public or private breastfeeding sustainability. The unfeasibility of prolonged public breastfeeding avoidance tactics often results in untimely breastfeeding discontinuation or the decision to avoid breastfeeding altogether (Scott et al., 2015; Stewart-Knox, Gardiner, & Wright, 2003).

More research is needed regarding infant feeding practices, beliefs, and attitudes towards breastfeeding behaviours among women living in Italy, given the lack of literature available in either Italian or English. Additionally, further exploration of Italian cultural influences on breastfeeding decision-making and societal perceptions may provide further insight into this community and translate to other areas of the world. This study explored women’s perceptions, attitudes, and experiences with breastfeeding living in Florence, Italy.

## Materials and methods

This study was part of a larger mixed-methods project conducted from June to July 2017. The larger study explored women’s reproductive health decision-making and experiences. This study explored women’s perceptions, attitudes, and experiences with breastfeeding living in Florence, Italy. Qualitative methodology allowed for a rich understanding of these women’s perceptions of and social norms surrounding breastfeeding.

Eligibility criteria included women of reproductive age (18–45 years), living in or around Florence, Italy, using the Italian healthcare system at the time of the study, and proficient in conversational English. Various recruitment strategies were used to increase participation. Printed recruitment flyers were placed throughout the Florence city centre detailing the purpose of the study and researcher contact information to schedule interviews. Additionally, a social media advertisement was created to reach a larger audience. Further, in-person participant recruitment was employed in which researchers approached women in public areas (e.g., libraries, cafes) to provide flyers and study information. Researchers also used snowball sampling (Berg & Lune, 2012), where participants suggested other women who may be eligible to participate, to increase participation levels. Women who participated in the survey portion of the study were prompted to provide personal contact information if interested in being contacted for a follow-up interview. Any contact information provided was kept separate from survey responses.

### Interviews

In-depth interviews were conducted (N = 44), each lasting approximately one hour, and took place in comfortable and convenient locations based on participant preferences (e.g., cafés, private offices). Participants provided informed consent to audio record the interview before beginning each interview. Interviews were recorded using the SoundNote iOS application and transcribed verbatim for subsequent data analysis. Following each interview, participants filled out an anonymous demographic form and were compensated with a 20 Euro gift card for their participation. Researchers utilized a semi-structured interview guide, allowing for purposeful questioning, conversation flexibility, and optimal participant-researcher rapport. The semi-structured nature of the interview allowed researchers to add, reorder, or modify questions based on the natural flow of conversation. Researchers probed on participant responses as needed to ensure questions were fully understood and adequately answered. The rapport building and flexible components of the semi-structured interview methodology assure participant responses are descriptive and in-depth. Additionally, it allowed participants to introduce new and relevant concepts during the interview process. Interviews began with general questions about each participant’s daily routine to increase comfort level and disclosure (Berg & Lune, 2012). Questions then investigated women’s perceptions of breastfeeding. Representative interview questions include: *Describe the breastfeeding culture in your community; Do most women choose to breastfeed; Do women breastfeed in public? Are there special places for women to go to breastfeed?*; and *How often do you see women breastfeeding in public in Florence?*. This range of questions allowed participants to discuss breastfeeding attitudes and norms personally and generally. Interviews continued until data reached theoretical saturation (Saunders et al., 2018) and study concepts were fully developed. Interviews provided insight into participants’ unique experiences and cultural norms related to breastfeeding practice, acceptance, and motherhood identity social norms.

### Participants

The mean age of the 44 participants was 31.7 ± 6.14 years (range = 19–45). Nearly all (*n = *41) women lived in Florence at the time of the study, while some (*n* = 2) lived in other cities within Tuscany, and one lived in a city outside of Tuscany. Women self-identified as heterosexual (*n* = 37), bisexual (*n* = 6), or homosexual (*n* = 1). Many participants had either initiated or completed college (*n* = 37), while some participants had completed high school or less (*n* = 7). Most participants were in a relationship (*n* = 30) or single (*n* = 9), and some were married (*n* = 5). The original sample included two women with children; however, they were excluded from this study as understanding societal attitudes towards breastfeeding was a primary focus. Further demographic information can be found in Table 1.

**Table 1.** Participant demographics

Variable (N = 44)	n
Gender	
Female	44
Age	
18 to 29 years	17
30 to 39 years	24
40 to 50 years	5
Sexual Orientation	
Heterosexual	37
Homosexual/Bisexual/Asexual/Intersex/Queer	7
Relationship Status	
Single	9
In a Relationship	30
Married	5
Education	
High School Diploma/GED or Lower	7
Education Beyond High School	37
Income	
Less than 30,000 Euro	19
30,000 to 69,999 Euro	17
70,000 Euro or more	2
Undisclosed	6
Current City of Residence	
Florence	41
Other Tuscan City	2
City Outside of Tuscany	1

Frequencies that do not sum to total represent missing data.

### Research team

Data were collected and transcribed verbatim by 15 undergraduate and graduate students participating in a research-based study abroad programme offered by a top-tier university in the Midwest, which is why all interviews were conducted in English. All students were trained in graduate-level qualitative research methodologies and immersed into the Florence community and culture for two months during the data collection period. Coding and analyses were completed by the second author, with a robust history of qualitative research, and one undergraduate student who participated in the study abroad. Authors utilized data tables and mind mapping for a strategic approach to analysis.

### Analyses

All interviews were transcribed verbatim along with any memos and observer comments to maintain reflexivity and identify emerging patterns. Content analysis within and across transcripts provided a basis upon which to determine the presence of participant words, phrases, and concepts to make meaning of the data (Hsieh & Shannon, 2005). This method of data analysis allowed researchers to identify and analyse emerging patterns and themes within the data (Corbin & Strauss, 2007). Participant words, phrases, and experiences provided codes throughout the data collection and analysis process (Corbin & Strauss, 2007). HyperRESEARCH 3.5.2., a qualitative data analysis software, was used to code, analyse, write and share memos, and maintain reflexivity throughout the analysis process. Researchers developed a line-by-line codebook based on initial readings of the data. Then, researchers completed line-by-line open coding. This iterative process allows for initial reflection on content and meaning established in the data. Following open coding, researcher completed axial coding to identify relationships among codes and to broader categories and patterns (Corbin & Strauss, 2007). Constant comparison between and within interview codes allowed researcher to identify emerging themes and build thematic categories. Researchers and research assistants regularly met throughout the data collection phase to discuss interview findings and explore emerging themes. Any discrepancy in coding was resolved through consensus.

### Ethical considerations

Purdue University’s institutional review board (Protocol #: 1611018435), with a letter of support from the Italian partner university, approved this study. The research conformed to all ethical principles for medical research on human subjects, per the Declaration of Helsinki (World Medical Association, 2013). Additionally, the study fulfilled all requirements for research, including: information, consent, confidentiality, and safety, and abided by ethical research principles cited by the Belmont Report, autonomy, beneficence, non-maleficence, and justice (National Commission for the Protection, 1979). Participants were adequately informed of the study and were notified of their right to withdraw participation at any point in the interview without explanation. Participants also provided both written and verbal informed consent to participate in the interview and be audio recorded (for transcription purposes). Upon transcription completion, interview audio files were destroyed. Demographics forms did not have a section for participant names, as the forms were used to provide de-identified information about the interview sample. Interview consent forms were kept separate from the data and demographics forms and kept in a secure, locked location.

## Results

Three overarching qualitative themes emerged from the in-depth interviews: 1) Breastfeeding trends and influences; 2) The role of identity and empowered choice; and 3) Perspectives on breastfeeding in public. Themes and corresponding participant quotations are summarized below.

### Breastfeeding trends and influences

Women generally felt positive and open towards breastfeeding, expressing it was a common and widely accepted practice throughout Italy that “Italian women normally do,” suggesting “it’s a good thing to breastfeed here in Italy.” These positive perspectives among the sample demonstrated beliefs that “people [in Italy] are definitely pro-breastfeeding.” Some participants argued breastfeeding is best because “the natural way is preferred.” Participants repeatedly emphasized the importance of employing the most natural methods of nourishment for babies, stating: “I don’t know anyone who didn’t want to [breastfeed], they do it in a natural way.” One woman stressed breastfeeding as the superior feeding method, stating formula should only be used as an absolute last resort for a mother, “if you really want to give them milk, then go for the formula. But, please try everything, even pumping, before giving up [breastfeeding]. You have to try your best.”

Other women contended a general consensus on breastfeeding prevalence did not exist. In these instances, participants believed breastfeeding practices “depends on the philosophy of the mother, there is not one particular, or, you know, mainstream sort of thinking.” Women in the sample noted differences “even among my friends, there are very different point of views on breastfeeding.” Participants provided various interpretations regarding the divide in breastfeeding preferences among mothers in Italy. One woman believed breastfeeding was, in fact, an uncommon practice, “I feel like I know more people who have done the formula feeding rather than breastfeeding.” Another woman stated the decision to breastfeed may depend on accessibility to childcare provisions and the mother’s breastfeeding availability and ability, “I think that’s more just a difference of how childcare [operates] in general. Some women take even up to three years off [of work to breastfeed], it depends.”

Participants noted breastfeeding was also influenced by professional opinions (i.e., advertisements, doctors). Some women expressed how the knowledge of professional opinions could decrease feelings of discomfort or embarrassment towards breastfeeding:

I saw on television once, I think like an advertisement, talking about the [breastfeeding] issue and [stating] this, of course, is something very beautiful and natural. So, women should not feel guilty or embarrassed [to breastfeed] because when the child needs to be [fed], it’s just time to do that.

Doctors emerged as the primary professional influence encouraging breastfeeding behaviours. One woman mentioned doctors begin discussing breastfeeding early in the pregnancy, “[doctors] do talk to you, before your birth, they talk about [breastfeeding].” Many women expressed favourable feelings towards breastfeeding because of the significance doctors place on it: “because it’s more important and the doctor says that breastfeeding [is] good for the children.” Another stated, “I will always choose breastfeeding because a lot of doctors say that is the best [and] the milk from the mother is the best nourishment you can actually give to your child.” Women also felt doctors’ expert opinions contributed to breastfeeding becoming a preferred, socialized behaviour, that “women prefer to [breastfeed] because it is very suggested from a doctor.”

Participants commonly mentioned an additional motivation to breastfeed was the positive health benefits for the baby. One woman alluded to this, stating this belief was commonly shared in Italy. She described, “I think that in Italy there is a strong culture about breastfeeding because […] if you breastfeed the baby for a lot of time, it’s better for the baby. They grow healthier.” Others agreed with breastfeeding was “for the health of the baby.” Some participants suggested breastfeeding yields mutual health benefits for mothers and babies. This was presented as an additional incentive to breastfeed, as one woman asserted, “the milk is good, but the milk of the mother is the best [for both]—absolutely.” Thus, participants identified social and health facilitators to breastfeeding.

### Role of identity and empowered choice

Effects of the motherhood identity on breastfeeding behaviours were repeatedly discussed during interviews. The motherhood identity, as described by participants, often represents a woman’s dominant identity after giving birth and speaks to social norms surrounding expectations of women and mothers. Participants viewed breastfeeding as one aspect of society’s expectations surrounding motherhood duties and responsibilities. One woman affirmed, “I think yes … [as a] woman, [she] is to breastfeed her child.” Another woman candidly agreed, “she [became] a mom, she has to behave like a mom.”

Mothers and the motherhood identity were highly respected in Italy, according to participants. These levels of respect were often associated with acceptability of breastfeeding behaviours and breastfeeding culture. One woman described Italians as being “quite maternal in that sense,” explaining, “I don’t think people would judge [breastfeeding], [we have] a lot of respect for mothers.” Another participant discussed the importance of family and how familial inclusion contributes to how breastfeeding is viewed:

It’s a good thing to breastfeed in Italy … family is so important, kids are so important, it’s really a thing I like [about] Italy. Whenever you go to the restaurant … also if you go to a wedding [you see women breastfeeding]. In [other cultures] it’s very separate, kids are not welcome in public. But here, [inclusion] is so much more important.

Some women explained even breastfeeding in public was respected and acceptable because it is part of being a mother. Although all participants were childless, they maintained the perception that because of its association with motherhood, breastfeeding should not be viewed as shameful or embarrassing. As one woman summarized, “even feeding the children in public places [should not be] taboo. It’s something that is a sweet moment [with a child.]” She continued, “it’s not really a problem at all,” suggesting the positive outlook on mothers who breastfeed their infants.

Other participants problematized the association between breastfeeding and motherhood identity. Some felt this placed too much pressure on women *to* breastfeed, suggesting a woman would be viewed as “less of a mother” if she decided against it. One participant detailed potential implications that could result from a lack of perceived and socially acceptable alternatives to breastfeeding and the pressure to breastfeed:

I haven’t [heard anything] saying if you don’t breastfeed, it’s not going to be the end of the world. And I find it really wrong because I think it really puts a lot of pressure on the mother, whether she decides [to or] not to [breastfeed] … She might need to go back to work, or she doesn’t feel like she wants to, or maybe she doesn’t [feel she] has any milk.

Another problem participants mentioned was the conflict between pressure to breastfeed and maintaining a career. Participants suggested women in Italy frequently had to choose between the children and career, “after a few months [when] you have to go back to work, you have to pull the milk out of the breast before. It’s probably not always very easy when you have a hundred other things to do.”

Some participants emphasized women should be allowed the freedom to choose whether or not to breastfeed without fear of judgement or concern of breaking motherhood identity expectations. One woman mentioned this empowered choice became prevalent beginning in the 1980s:

I think that at that time women were not breastfeeding. But it was kind of connected to sexual freedom and the fact that they were not … they didn’t have to do that. Like my mom didn’t breastfeed me nor my sister.

Another woman agreed with this increased frequency of freedom in breastfeeding decision-making, “the ones who want to [breastfeed] … they feel free to do it … it’s becoming normal to choose not to do it and it is becoming normal if you choose to do it—to do it with freedom.” Other participants specified reasons why some women choose not to breastfeed, including “it is quite normal choosing not to breastfeed […] for aesthetic or career issues,” while another offered an example aesthetic concern, “a lot of mothers just choose not to [breastfeed] because they don’t want their breasts to sag.”

### Perspectives on breastfeeding in public

When asked about their perspectives surrounding breastfeeding in public, participants offered varied responses. Most agreed breastfeeding in public was viewed as a common practice and expressed “it’s not uncommon to see women sitting outside in public breastfeeding […] we might be at dinner with friends that have a baby and, you know, at the table they just breastfeed. It’s very normal to just be there.” Another woman agreed, “[breastfeeding] is a very natural thing, without exposing too much but not hiding it at the same time. It’s a very natural thing that a woman can do with [her] child.” One participant clarified the acceptability of exposed breasts in public for breastfeeding purposes, “I think in [the] Italian society, it’s more frowned upon to [just have] your cleavage out, [rather than having] your cleavage out because of a child.” She went on to provide a comparison to U.S. perceptions of public breastfeeding, “it is different than in the US, because I know in the U.S. it’s like ‘ahhh’ and people have those blankets and they hide the child under the blanket. No, not here. Here, people [just] breastfeed.” Other women supported this, expanding on the perceived “American view” of public breastfeeding and rejecting its occurrence in Italy. One woman recounted a time when her friend breastfed publicly, “she breastfed in front of my boyfriend, you know, like with her boob out. Like it’s okay, I mean it’s breastfeeding, it’s not something sexy or anything.” Another participant agreed, “I don’t think we see [breastfeeding] as sexualized at all, it’s the opposite. There’s nothing sexual. It’s seeing a baby sucking on their mother’s breast.”

Other participants felt it was uncommon to see public breastfeeding suggesting “people don’t breastfeed in public here. I don’t remember seeing it, like at all.” Another participant generalized to Italy, “it’s something you never see in Italy—very, very, rarely.” Another woman commented on the irony of the social norm against breastfeeding, “it’s really weird because here you will go to the beach and see a lot of boobs … that’s why we just laugh [because when it comes to breastfeeding] it’s like a social norm not to, I guess.” Although, some participants who did report seeing breastfeeding in public felt this practice was only acceptable if mothers have no other option. One woman affirmed, “if you really need to [breastfeed], maybe you can do it. But it’s really not [accepted] … it’s strange.” Another woman offered a bit of insight into its unacceptability:

Yeah, if you don’t have a choice, you have to [breastfeed in public]. Maybe it’s better not [to breastfeed] in restaurants or something like that […] because maybe you don’t want to see another woman’s breast while you are eating. It’s not [necessarily that we] don’t want to see that, it’s more [we] don’t like [to see that].

Another participant agreed with this perspective, “so people are kind of [like], ‘why is she feeding her son here?’ I mean, he needs to eat but … ” Despite these participants’ perceptions, it remains difficult to determine breastfeeding in public was uncommon due to social stigma or because breastfeeding rates are generally low.

A few women expressed blatant disapproval of public breastfeeding behaviours, stating, “I think it could be better for all to breastfeed … not in public. In private.” Another woman noted her negative opinions, “I have terrible opinions about it, I’m aware. I’m against breastfeeding in public.” One woman offered one explanation for this view, “si, [I am against it] because I think, even if it’s natural, it’s not so beautiful to see.” Thus, appearance and discomfort appeared to affect her attitudes towards public breastfeeding. Some participants felt even some mothers would agree and condemn public breastfeeding behaviours due to feelings of embarrassment: “maybe [mothers] feel more uncomfortable in public because they are looked at more.” This participant conjectured discomfort was associated more with others’ attention and potential disapproval of public breastfeeding. Social norms varied and appeared to impact breastfeeding behaviour and acceptance.

## Discussion

This study explored women’s perceptions, attitudes, and experiences with breastfeeding living in Florence, Italy. Three themes were identified discussing current breastfeeding trends and influences, the role of identity and empowered choice, as well as perspectives on public breastfeeding behaviours. Research findings contribute to the limited—yet growing—body of literature surrounding breastfeeding practices and perspectives in an Italian cohort (Banderali, Riva, Scaglioni, Agostoni, & Giovannini, 2003; Farchi, Asole, Chapin, & Lallo, 2016; Giovannini et al., 2004; Kambale, 2011; Lauria et al., 2016; Quintero Romero et al., 2006; Scott et al., 2015; Synnott et al., 2007). Results provided insight into breastfeeding behaviour influences in Italy, social norms surrounding motherhood identity and the impact of power of choice on breastfeeding decision-making.

The existing acceptability and propensity to breastfeed expressed by women in our sample bodes well for future efforts towards expanding breastfeeding knowledge and encouragement. Although past literature on breastfeeding influences in Italy is scarce (Bellù & Condò, 2017; Di Mattei et al., 2016; Hawkins et al., 2007; Scott et al., 2015), results are consistent with previous literature in the EU and US demonstrating main influences for breastfeeding behaviours are recommendations from doctors and health professionals, positive health benefits for baby, and the perception of breastfeeding as the “most natural” infant feeding method (Bagci Bosi et al., 2016; WHO, 2017). This suggests consistency in the understanding of health benefits, the impact of influential variables, and the breastfeeding behaviours and beliefs across the US, other countries in the EU, and Italy. However, despite the policies and other structural efforts enacted to encourage breastfeeding (i.e., parental leave, BFHI, Innocenti Declaration, public breastfeeding acceptance, etc.), participants still perceived barriers to breastfeeding, indicating a lack of awareness of available resources. Better promotion and publicity of these available provisions may be necessary to inform women and cultivate breastfeeding empowerment.

The Italian cultural value of motherhood surfaces significant implications for breastfeeding behaviours and decision-making. The motherhood identity as the dominant role emphasizes the importance and respect placed on women who bear children in Italy (Marini-Maio & Faleschini Lerner, 2018). However, there is an apparent paradox associated with this idea. The reverence of motherhood in the Italian culture may result in societal pressure on mothers (Marini-Maio & Faleschini Lerner, 2018), creating unrealistic expectations related to breastfeeding decision-making. Participants suggested mothers who deviated from Italy’s breastfeeding norm were violating their role as a mother, thus tainting their motherhood identity. Similarly, sustaining a career after birth was also often socially criticized because it meant the mother would lack the ability to stay at home and breastfeed her infant. This pressure placed on mothers limits the empowerment of choice in breastfeeding decisions, resulting in a complicated interplay of power and powerlessness (Giorgio, 2015). Though the motherhood role assumes power in care and decision-making for the infant, this powerlessness is evident through unsupportive societal expectations towards mothers who decide against breastfeeding, expressed even by women in this sample who had not given birth. Regardless of the various contexts in which a woman would choose not to breastfeed (e.g., cannot produce milk, desire to maintain a career), the shame component persists.

The varying public breastfeeding perceptions expressed by participants further perpetuates shame. Results displayed mixed reviews surrounding perceptions of public breastfeeding; participants’ support existed along a spectrum (Scott et al., 2015). In some cases, breastfeeding in public was perceived as acceptable because it was a natural part of having an infant. However, others expressed they were particularly against breastfeeding in public. Many of these were people who agreed breastfeeding was a positive thing for mothers to do, but “even if it’s natural, it’s not so beautiful to see.” Again, this references the paradoxical idea of pressure on mothers. Breastfeeding is seen as something positive, but the caveat remains. These social and cultural attitudes undermine maternal confidence, which may in turn negatively affect current or future breastfeeding decision-making (Grant, Morgan, Mannay, & Gallagher, 2019; Rollins et al., 2016).

Our results support the limited literature available and contribute insights to the growing body of literature surrounding breastfeeding in Italy (Bagci Bosi et al., 2016; Di Mattei et al., 2016; Farchi et al., 2016; Giovannini et al., 2004; Kambale, 2011; Lauria et al., 2016; Scott et al., 2015; Yngve & Sjöström, 2001). Results also provide practical recommendations for future exploration and application. In particular, social marketing campaigns and public health initiatives (Lubold, 2017) related to breastfeeding decision-making empowerment may improve women’s autonomy and the acceptability of their choices. Social marketing incorporates interventions, campaigns, and other marketing tools to improve societal, communal, and health issues, resulting in a shift in behaviour and overall social norms (Dibb, 2014). Individuals have an innate propensity to adopt social norms regarding what they are exposed to or to have negative outlooks on what they perceive as violating social norms (Ostrom, 2014). Thus, these contradicting perceptions appear to limit the prevalence and acceptability of breastfeeding in both public and private settings. Previous breastfeeding promotion social marketing campaigns exist; however, these often focus on the health benefits of breastfeeding (Cartwright, Atz, Newman, Mueller, & Demirci, 2017; Hussein, Manna, & Cohen, 2014; Pérez-Escamilla, 2012). These methods, though effective in supporting breastfeeding as the best infant feeding method, fail to address the empowered choice component—proving less effective in addressing all the benefits and costs women may perceive associated with breastfeeding choice. If women feel their lived experiences are not reflected in these initiatives, they may not be successful in influencing behaviour and social norms. We suggest the utilization of social marketing campaigns with a focus on breastfeeding choice *support*, whether it is done in private, public, or not at all. Bringing breastfeeding support imagery and messaging into public consciousness by understanding perceptions prior to pregnancy or breastfeeding decisions can invoke breastfeeding audiences and may contribute to behaviour normalization (Giles, 2018). Utilizing these empowerment strategies may alter how the public views breastfeeding decision-making, shift the social norms of acceptable motherhood, and potentially result in the minimization of breastfeeding barriers and shaming behaviours (Aldoory, Braun, Maring, Duggal, & Briones, 2015; Grant, 2016).

### Strengths and limitations

This study is the first of its kind to utilize a qualitative approach to explore breastfeeding behaviours, influences, and perspectives in Italy and publish in English. A previously conducted case study informed the interview guide, incorporating expert opinions and content analysis. Cultural appropriateness of the instrument was reviewed and approved by Italian experts. All in-depth interviews were conducted in English, which may have limited participation, and may have resulted in misinformation or miscommunication, and presented some interpretation bias. Future research should explore this content in both Italian and English. The majority of interview participants were recruited from their workplaces in or near the Florence city centre; therefore, generalizability is limited. However, the study methodology provides a basis upon which to explore breastfeeding perspectives in other contexts, specifically those with similar cultural values (i.e., conservative, family-oriented, religious), suggesting the transferability of the work (Houghton, Casey, Shaw, & Murphy, 2013). Self-identifying as Italian or an Italy native was not one of the study criteria, nor was it captured on the demographic information form, which could have introduced variability in experiences and perceptions. Additionally, this research was conducted in Tuscany, a region in Italy with numerous baby-friendly initiatives and strong breastfeeding support programmes. Despite this, our findings captured a strong negative public breastfeeding perception and social stigma. Further research should explore the impact of these programmes on breastfeeding perceptions and attitudes. No women in the current sample reported having children of their own; however, as questions primarily focused around societal attitudes towards breastfeeding, the nulliparous sample was able to offer unique perspectives related to perceived social norms of breastfeeding. Dependability was represented within the study as women shared similar attitudes towards breastfeeding, indicating a convergence of logic and general understandability among participants (Lincoln & Guba, 1985). Interviews were conducted by several research assistants trained in graduate-level methodologies and immersed in the community as part of an extended study abroad experience. The in-depth individual interview methodology, coupled with investigator triangulation, or using multiple researchers to mitigate any bias or influence, contributes to study credibility (Carter, Bryant-Lukosius, DiCenso, Blythe, & Neville, 2014). The team met regularly and discussed interview experiences and emerging data trends to inform any necessary protocol adjustments and allowed the primary investigator to monitor coding and data reliability. Researcher memos, all codebook iterations, and data from all stages (i.e., raw data, audio, transcriptions, coded data) related to this study remain preserved, supporting confirmability of the research (Lincoln & Guba, 1985). Despite the limitations, our study provides a meaningful contribution to the breastfeeding literature and offers novel information regarding breastfeeding trends, influences, and perceptions among a sample of women living in Italy.

## Conclusions

Breastfeeding represents a behaviour impacted by social norms of acceptability. Findings support limited available literature and contribute insights surrounding breastfeeding in Italy. This study explored women’s perceptions, attitudes, and experiences with breastfeeding living in Florence, Italy. Reverence of motherhood in the Italian culture appeared to result in societal pressure, as mothers who deviated from Italy’s breastfeeding norm were viewed as “violating” their role as a mother. Career resumption after birth was an additional social criticism as it translated to insufficient breastfeeding duration. Thus, perceived pressure on mothers limited empowerment, resulting in an interplay of power and powerlessness over breastfeeding choice, and an air of social and personal shaming. This breastfeeding paradox suggests a need for improved messaging strategies that seek to empower women in their breastfeeding choices. Future research should formulate and implement evidence-based social marketing campaigns focusing on breastfeeding choice *support*, whether mothers choose to do so in private, public, or not at all. Additionally, messaging should allude to existing breastfeeding policies and provisions to better inform mothers of their rights and the resources available to encourage and support breastfeeding initiation and sustainability. Breastfeeding support imagery and messaging should also consider the various goals women may also choose to prioritize along with motherhood (i.e., career investment, aesthetic, etc.), as this may appeal to breastfeeding audiences and contribute to behaviour normalization. Utilizing social marketing strategies with a focus on empowerment may alter public views surrounding breastfeeding decision-making, shift social norms of “acceptable motherhood,” and potentially minimize perceived breastfeeding barriers and shaming behaviours.

## Data Availability

The datasets used and analyzed for the current study are available from the corresponding author upon reasonable request.
